# pH-Triggered Sheddable Shielding System for Polycationic Gene Carriers

**DOI:** 10.3390/polym8040141

**Published:** 2016-04-14

**Authors:** Jialiang Xia, Huayu Tian, Jie Chen, Lin Lin, Zhaopei Guo, Bing Han, Hongyan Yang, Zongcai Feng

**Affiliations:** 1 School of Chemistry and Chemical Engineering, Lingnan Normal University, 29 Cunjin Road, Zhanjiang 524048, China; hbzlz@yeah.net (B.H.); hongyan7910@126.com (H.Y.); fengzongcai921@163.com (Z.F.); 2 Key Laboratory of Polymer Ecomaterials, Changchun Institute of Applied Chemistry, Chinese Academy of Science, 5625 Renmin Street, Changchun 130022, China; chenjie@ciac.ac.cn (J.C.); linlin@ciac.ac.cn (L.L.); wfgzp@ciac.ac.cn (Z.G.); 3 Development Center for New Materials Engineering & Technology in Universities of Guangdong, Zhanjiang 524048, China

**Keywords:** gene delivery, shielding system, pH-sensitive

## Abstract

For improving the therapeutic efficiency of tumors and decreasing undesirable side effects, ternary complexes were developed by coating pH-sensitive PEG-*b*-PLL-*g*-succinylsulfathiazole (hereafter abbreviated as PPSD) with DNA/PEI polyplexes via electrostatic interaction. PPSD can efficiently shield the surface charge of DNA/PEI. The gene transfection efficiency of ternary complexes was lower than that of DNA/PEI at pH 7.4; however, it recovered to the same level as that of DNA/PEI at pH 6.0, attributed to the pH-triggered release of DNA/PEI from ternary complexes. Cell uptake results also exhibited the same trend as transfection at different pH values. The suitable ability for pH-triggered shielding/deshielding estimated that PPSD demonstrates potential as a shielding system for use in *in vivo* gene delivery.

## 1. Introduction

Cancer is one of the most lethal diseases worldwide. Gene therapy has emerged as a promising strategy for treating cancer and has been developed rapidly [1]. The safe and efficient delivery of nucleic acids remains a challenge as several barriers have to be overcome within the delivery pathway. In addition, delivery systems designed for *in vivo* systemic administration must overcome nonspecific interactions of polyplexes with cells of the immune system and blood plasma proteins during their transport to target cells [2]. These obstacles are successfully suppressed by the modification of gene carriers with PEG [3,4] or other hydrophilic polymers [5,6,7].

Different pH values of the microenvironment between tumor and normal tissues have attracted immense attention [8,9,10,11]. For improving the stability in blood circulation and providing environmental targeting ability, different pH-sensitive shielding systems for cationic gene delivery were developed. Most of these pH-sensitive materials exhibit negative charges at the pH of normal tissues; hence, positively charged gene delivery cargoes can be enclosed and protected via electrostatic interaction. Moreover, at the pH of tumor tissues, these materials change to neutral or positive charge, through which they recover the positive charge of gene delivery cargoes and enhance cell uptake and gene transfer efficiency. A kind of shielding–deshielding system composed of oligo-sulfonamide derivatives was developed [12,13,14,15]. The oligo-sulfonamide segment exhibits changes of negative to neutral charge with the change in the surrounding pH from base to acid. Another pH-sensitive, charge-conversional polymeric shielding system was developed by grafting multiple carboxylic groups to cationic polymers via a degradable amide bond [7,16,17,18,19,20]. Under basic conditions, the shielding system exhibits negative charge, attributed to the carboxyl groups; however, under acidic conditions, the amide bond degrades and releases positively charged amino groups. Zwitterionic copolypeptide also shows a charge conversion property with the change of pH. The zwitterionic copolypeptide thus developed with negative charge at physical pH can act as a shielding system for shielding positively charged polyplexes, and the polyplex surface zeta potential can change from a negative to nearly positive pH value of 6.9 [21,22,23,24]. Charge-shielding systems utilized for cationic gene carriers have also been reported to be formed by other polymers with multiple carboxylic groups, such as polyglutamic acid [25,26,27,28] and hyaluronic acid [29]. All these polymers develop stable ternary particles with DNA/polycations at physiological pH and then deshield and release the DNA/polycations for transfection when the ternary complexes reach the acid tumor site.

In the study, biodegradable, pH-sensitive mPEG-*b*-PLL-*g*-succinylsulfathiazole (hereafter abbreviated as PPSD) was prepared and characterized as a shielding system for PEI. A DNA/PEI/PPSD ternary complex as well as distinct transfection behavior at pH 7.4 and 6.0 was investigated.

## 2. Experimental Section

### 2.1. Materials

Branched PEI (*M_W_* = 25 kDa, PEI-25K), ethidium bromide, and calf thymus DNA were purchased from Sigma-Aldrich Co. LLC. (Shanghai, China). The BCA protein assay kit was purchased from Pierce (Rockford, IL, USA). Luciferase plasmid (pGL3-control), cell lysate, and the Luciferase Reporter Gene Assay Kit were purchased from Promega (Mannheim, Germany). mPEG-NH_2_ (*M*_n_ = 3k) was purchased from Meryer Chemical Technology Co., Ltd. (Shanghai, China). All other chemicals were purchased from Sinopharm Chemical Reagent Co. Ltd. (Shanghai, China) without further treatment.

### 2.2. Synthesis and Characterization of PPSD

Grafting succinylsulfathiazole (SSD) to mPEG-*b*-PLL (3k–2k) by NHS/DCC condensation yielded PEG-*b*-PLL-*g*-SSD (abbreviated as PPSD). SSD was prepared by reaction between sulfadimethoxine (SD) and succinic anhydride.

The pH sensitivity of PPSD was investigated by pH titration. Briefly, PPSD was dissolved in water at a concentration of 1 mg/mL. The solution pH was adjusted to 9.5 using a 0.1 M·NaOH solution. Then, the solution was titrated using a 0.1 M HCl solution in increments of 5 μL, and the pH of PPSD solution, volume of HCl added, and zeta potential of PPSD were monitored.

### 2.3. Preparation and Characterization of DNA/PEI/PPSD Ternary Complexes

The DNA/PEI/PPSD ternary complexes were prepared by mixing the PPSD solution with DNA/PEI nanoparticles at different weight ratios. Briefly, DNA/PEI polyplexes were prepared at a weight ratio of 1:1 at room temperature with a DNA concentration of 10 μg·mL^−1^. Then, a specific amount of PPSD was mixed with DNA/PEI polyplexes, and then DNA/PEI/PPSD ternary complexes were obtained. The final concentration of DNA in ternary complex solution was 5 μg·mL^−1^.

The zeta potential and particle size of the ternary complexes in the aqueous solution were measured using a zeta potential/particle size analyzer (Brookhaven BI-90Plus, New York, NY, USA) at room temperature. The development of ternary complexes was characterized by a gel retardation assay.

### 2.4. Cytotoxicity Assay

HeLa cells were seeded at 1.0 × 10^4^ cells/well in 96-well plates and then cultured for 24 h with different amounts of PPSD. The concentration of PSD cultured with HeLa cells was 10 to 400 μg·mL^−1^. The relative cytotoxicity of PPSD was assessed by the methyl thiazolyl tetrazolium (MTT) assay against HeLa cells. Cell viability (%) = (*A*_sample_/*A*_control_) × 100, where *A*_sample_ and *A*_control_ represent the absorbance values of the sample and control wells, respectively.

### 2.5. In Vitro Gene Transfection at Different pH Values

An *in vitro* gene transfection experiment was performed using HeLa cells at different pH values. Briefly, HeLa cells were seeded and cultured for 24 h, and then the medium was replaced with fresh DMEM medium at pH 7.4 or 6.0 containing pGL3-control/PEI or pGL3-control/PEI/PPSD complexes. The cells were incubated for 4 h at either pH 7.4 or 6.0. Then, the medium was replaced with fresh DMEM medium of pH 7.4 and incubated for an additional 44 h. The gene transfection efficiency was expressed as the luciferase expression per milligram of protein.

### 2.6. Cellular Uptake at Different pH Values

The cell uptake behavior of the DNA/PEI polyplexes and DNA/PEI/PPSD ternary complexes in HeLa cells at both pH 7.4 and 6.0 was observed by confocal laser scanning microscopy (CLSM, LSM 780, Carl Zeiss Inc., Jena, Germany) using CY5-labeled DNA. HeLa cells were seeded and incubated for 24 h in six-well plates. The medium was replaced with DMEM medium (pH 7.4 or 6.0) containing DNA/PEI or DNA/PEI/PPSD complexes. The cells were incubated for further 2 h at either pH 7.4 or 6.0 and then fixed by paraformaldehyde (4% in PBS) for 10 min at room temperature. The cellular uptake of complexes was visualized by CLSM after the nucleus was stained with DAPI.

For flow cytometric analysis, HeLa cells were seeded in six-well plates at 2.0 × 10^5^ cells per well in 2 mL of complete DMEM medium and cultured for 24 h. The medium was replaced with DMEM medium (pH 7.4 or 6.0) containing DNA/PEI or DNA/PEI/PPSD complexes. The cells were incubated with the complexes for 2 h at either pH 7.4 or 6.0 and washed two times with PBS (pH 7.4). The cells were detached using 0.25% trypsin and resuspended in 300 μL PBS. Finally, the cellular uptake efficiency was evaluated using a BD FACS Calibur flow cytometer (BD Bioscience, San Joe, CA, USA).

## 3. Results and Discussion

### 3.1. Synthesis and Characterization of PPSD

**Scheme 1:**
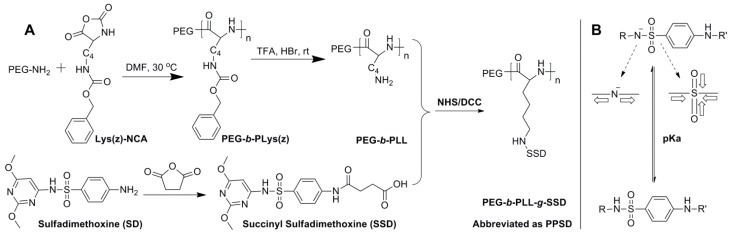
Synthesis of PPSD (**A**) and the pH-sensitive mechanism of sulfonamide units (**B**).

Scheme 1A shows the synthetic route of PPSD, while the ^1^H NMR and FT-IR results were shown in Figures S1–S4 (Supplementary Materials). The peaks at 2.50~2.60 ppm indicated the succinic group had been conjugated to SD (Figure S1). The ^1^H NMR measurements confirmed the synthesis of PEG-PLys(z) and PEG-PLL, and the degree of polymerization of the PLL segment was also confirmed by ^1^H NMR in Figure S2, that is 16. The degree of grafting of SSD is nearly 100% (Figure S3), and the structure of PPSD was also confirmed by FT-IR spectra (Figure S4).

Scheme 1B shows the mechanism of the pH sensitivity of sulfonamide derivatives. Sulfadimethoxine is a weak acid, and the hydrogen atom of the amide nitrogen can be readily ionized at basic pH because the highly electron-attracting sulfonyl group draws electrons away from the nitrogen atom, resulting in ionization and a negative charge. In other cases, sulfadimethoxine is neutral at acidic pH.

**Figure 1:**
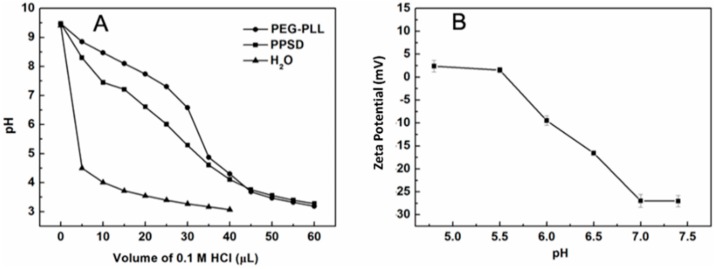
pH sensitivity of PPSD: (**A**) pH buffer capacity titration of PPSD; (**B**) Zeta potential of PPSD solution at different pH.

Acid-base titration experiments were conducted for PPSD for evaluating the buffering capacity. As can be observed in Figure 1A, PPSD exhibited a buffer capacity similar to that of PEG–PLL. The results obtained from the zeta potential of the PPSD solution at different pH values indicated that PPSD is negatively charged at higher pH but changes to neutral at a lower pH of 5.5. As described before, the pH-triggered ionization-deionization property of SD may render intelligent shielding/deshielding properties to PPSD: PPSD can shield the positive charge of DNA/PEI polyplexes at high pH and release polyplexes at low pH.

### 3.2. Characterization of Ternary Complexes

**Figure 2:**
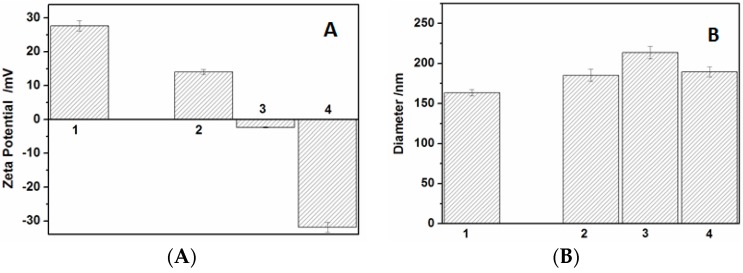
Surface charge (**A**) and particle size; (**B**) characterization of DNA/PEI/PPSD ternary complexes at pH 7.4. Lane 1 to 4, DNA/PEI/PPSD at ratios (*wt/wt/wt*) of 1:1:0, 1:1:8, 1:1:16, and 1:1:32, respectively.

The binary DNA/PEI polyplexes exhibited a positive surface charge (+27.7 mV) with a particle size of 163 nm at pH 7.4. After shielding with PPSD at different weight ratios, the surface charge of the DNA/PEI/PPSD ternary complexes decreased to neutral and even to negative (−31.2 mV) (Figure 2A), and the particle size predominantly was no greater than 210 nm (Figure 2B).

**Figure 3:**
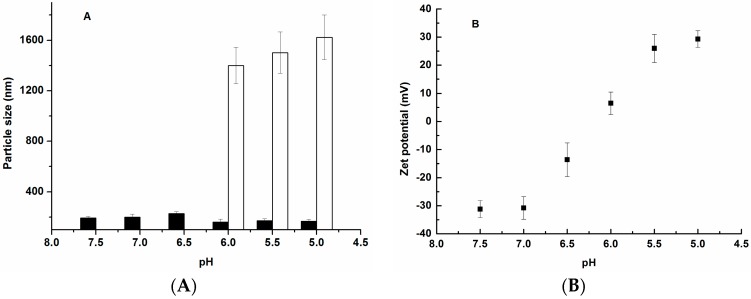
Particle size (**A**) and zeta potential (**B**) of DNA/PEI/PPSD (1:1:32) ternary complexes at various pHs. The solid bars could be the DNA/PEI complex and the open bars may be the aggregates of neutral PPSD in the (**A**).

After determining the particle size and zeta potential of ternary complexes in different PPSD/PEI/DNA mass ratios, we selected the mass ratio of PPSD:PEI:DNA as 32:1:1 complexes to investigate the particle size and zeta potential at various pHs (Figure 3). As the data show, from pH 7.5 to 6.5 unimodal particle distribution was observed, indicating PPSD complexed with DNA/PEI; then pH 6.0 to 5.5 showed bimodal particle distribution, and the solid bars could be the DNA/PEI complex and the open bars may be the aggregates of neutral PPSD. The zeta potential study indicates a similar trend, where the particles show a negative zeta potential between pH 7.5 and 6.5 which indicates complete complexation of the PPSD; the positive potential from pH 6.0 to 5.5 indicates decomplexation. These data indicated that the acidic condition should trigger the disassembly of the ternary complex.

**Figure 4:**
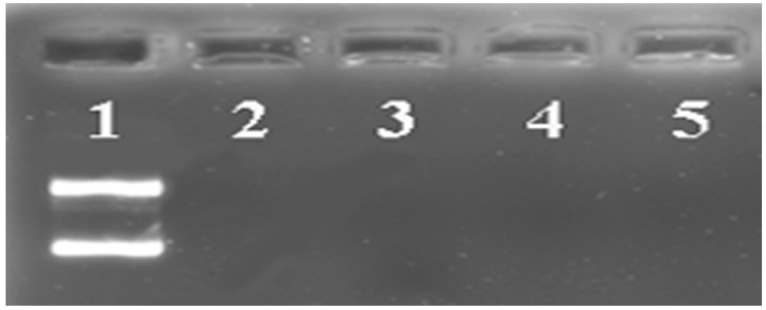
Gel retardation assay. Lane 1: DNA; lane 2 to 5, DNA/PEI/PPSD at ratios (*wt/wt/wt*) of 1:1:0, 1:1:8, 1:1:16, 1:1:32, respectively.

For further evaluating the stability of ternary complexes, the gel retardation assay was carried out. From the results obtained by the gel retardation assay, all ternary complexes were retarded, and the ethidium-bromide-intercalated DNA was not detected in the lanes (Figure 4). This result indicated that PPSD does not destroy the structure of the DNA/PEI polyplexes. All the above-mentioned results suggested that PPSD efficiently shields the DNA/PEI polyplexes and that the shielding degree is under control.

### 3.3. Cytotoxicity Assay, In Vitro Transfection, and Cellular Uptake Assay

**Figure 5:**
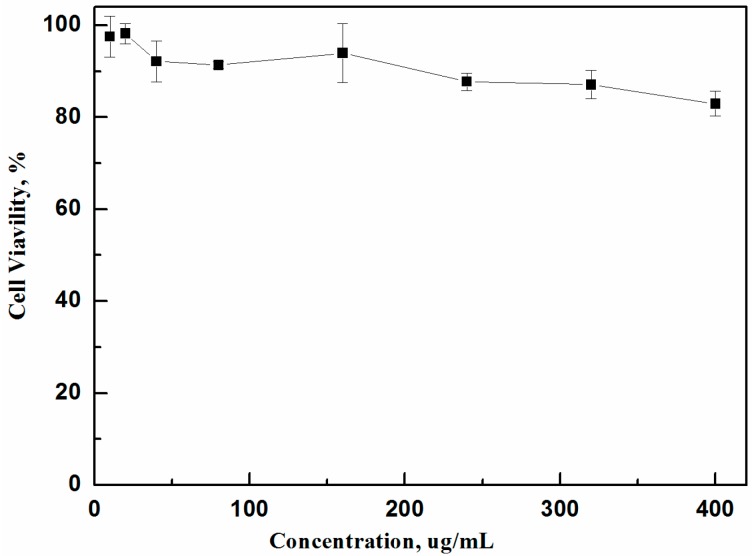
Cytotoxicity of PPSD.

The cytotoxicity of PPSD was investigated using HeLa cells by the MTT-based assay (Figure 5). The cell viability was at least 90% at the test concentrations. Results exhibited good biocompatibility of PPSD.

**Figure 6:**
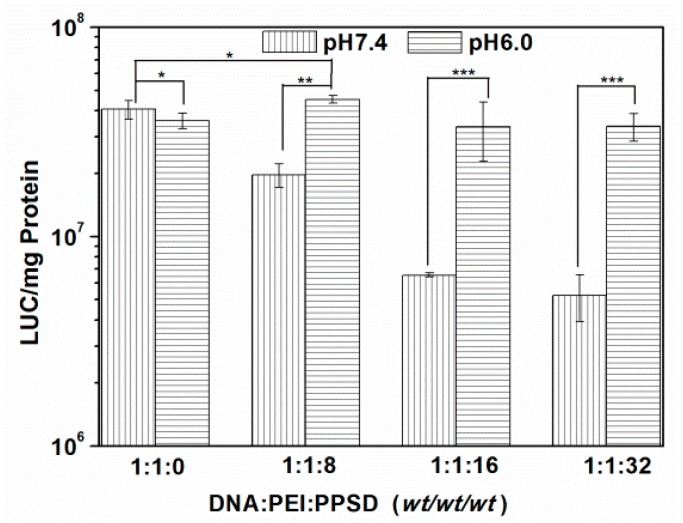
Transfection efficiency of the ternary complex at pH 7.4 and 6.0 in HeLa cells. *, *p* > 0.05; **, 0.01 < *p* < 0.05. ***, *p* < 0.01.

For mimicking the normal and tumor extracellular acid environments, the gene transfection activities of the DNA/PEI and DNA/PEI/PPSD complexes were evaluated using HeLa cells at either pH 7.4 or 6.0 (Figure 6). Gene transfection rapidly decreased with increasing amounts of PPSD in ternary complexes at pH 7.4. This low efficiency was mainly attributed to the shielding of the surface positive charges of the DNA/PEI polyplexes by PPSD. When the ternary complexes were treated with acidic buffer (pH = 6.0) during transfection, the transfection efficiency significantly recovered to that of the DNA/PEI level, attributed to the pH-trigged ionization/deionization behavior of PPSD, because as compared to negatively charged nanoparticles, positively charged nanoparticles adhere to the negatively charged cell membrane and are internalized by cells.

**Figure 7:**
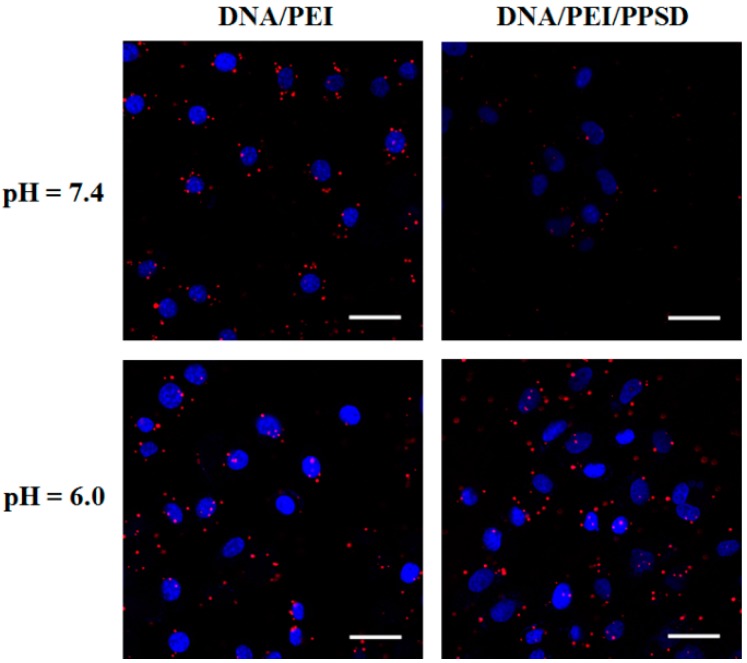
CLSM images of HeLa cells incubated with DNA/PEI (*wt*/*wt* = 1:1) or DNA/PEI/PPSD (*wt*/*wt*/*wt* = 1:1:32) at pH 7.4 or 6.0. DAPI, cell nucleus (blue); CY5-pDNA (red). Scale bars = 20 μm.

For investigating the recovery of the gene transfection of ternary complexes by treatment in acidic DMEM medium, the cell uptake of DNA/PEI and DNA/PEI/PPSD complexes under different pH conditions was assessed by CLSM. As shown in Figure 7, the DNA/PEI/PPSD ternary complex at a mass ratio of 1:1:32 was used as an example. From the fluorescence images, only marginal red fluorescence was observed at pH 7.4. The invariable low internalization effect was obviously attributed to the negative charge shielding of PPSD. In comparison, CY5-pDNA was effectively internalized into the cells at pH 6.0.

The negatively charged zeta potential and low gene transfection efficiency of ternary complexes at pH 7.4, as well as the recovered gene transfection by treatment with acidic DMEM at pH 6.0, indicated sufficient positive charge shielding, attributed to PPSD at pH 7.4, and deshielding at pH 6.0. The enhanced intracellular uptake was mainly attributed to the pH-trigged deshielding behavior of PPSD at pH 6.0. After deshielding, the positively charged surface of the DNA/PEI polyplexes was re-exposed, thereby enhancing the intracellular uptake of the complexes. Overall, cellular uptake studies clearly demonstrated that the negatively charged nanoparticles decrease internalization at pH 7.4, whereas the positively charged particles sharply enhance internalization at pH 6.0.

**Figure 8:**
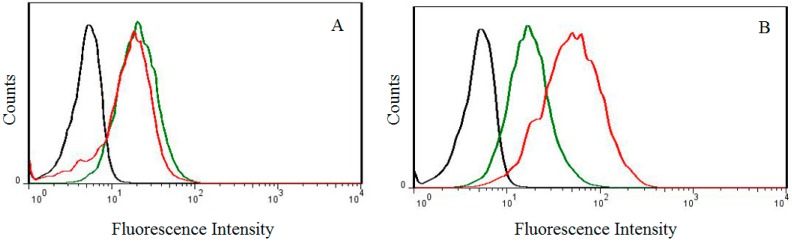
Cellular uptake of DNA/PEI (1/1) (**A**) and DNA/PEI/PPSD (1/1/32); (**B**) at different pH values in HeLa cells (gray, control; red, pH 7.4; green, pH 6.8) (All complexing ratios are expressed as weight ratios).

Intracellular uptake was further monitored by flow cytometric analysis using HeLa cells, which were cultured using the DNA/PEI or DNA/PEI/PPSD complexes for 4 h at either pH 7.4 or 6.0. As shown in Figure 8, the fluorescence peaks for the DNA/PEI/PPSD complex remarkably shifted to the right at pH 6.0, indicating that the internalization efficiency significantly improves in comparison to that of the cells cultured at pH 7.4. The differences in endocytosis efficiency may indicate that PPSD shielding can achieve a significantly enhanced gene transfection efficiency in an acidic tumor microenvironment.

## 4. Conclusions

In summary, PPSD was developed as a novel pH-responsive shielding system. PPSD can efficiently shield DNA/PEI polyplexes at pH 7.4. The DNA/PEI/PPSD ternary complexes exhibited transfection efficiency conversion from pH of 7.4 to 6.0 based on the pH-sensitive shielding/deshielding property of PPSD. Overall, PPSD demonstrates promise as a tumor microenvironment–sensitive gene delivery shielding system for antitumor therapy.

## Supplementary Materials

Supplementary materials can be found at www.mdpi.com/2073-4360/8/4/141/s1.
