# Supplementary material for: pH-Triggered Sheddable Shielding System for Polycationic Gene Carriers
Source: Polymers (Basel). 2016 Apr 14;8(4):141. doi: 10.3390/polym8040141 (PMC6432142; doi:10.3390/polym8040141)
Supplement: Supplementary file 1 [file polymers-08-00141-s001.pdf]

Supplementary Materials: pH-Triggered Sheddable Shielding System for Polycationic Gene Carriers

Jialiang Xia, Huayu Tian, Jie Chen, Lin Lin, Zhaopei Guo, Bing Han, Honyan Yang and Zongcai Feng

Synthesis of Succinylatedsulfonamide (SD), PEG-PLL and PEG-*b*-PLL-*g*-SSD (PPSD)

In a flame-dried flask, sulfathiazole, Succinic anhydride and pyridine were dissolved in 60 mL of anhydrous THF at molar ratio of 1:50:50, and stirred for 72 h at 60 °C. Then the product mixture was precipitated with an excess amount of water under vigorous stirring. The purified succinylatedsulfonamide (SSD) was obtained as white solid.

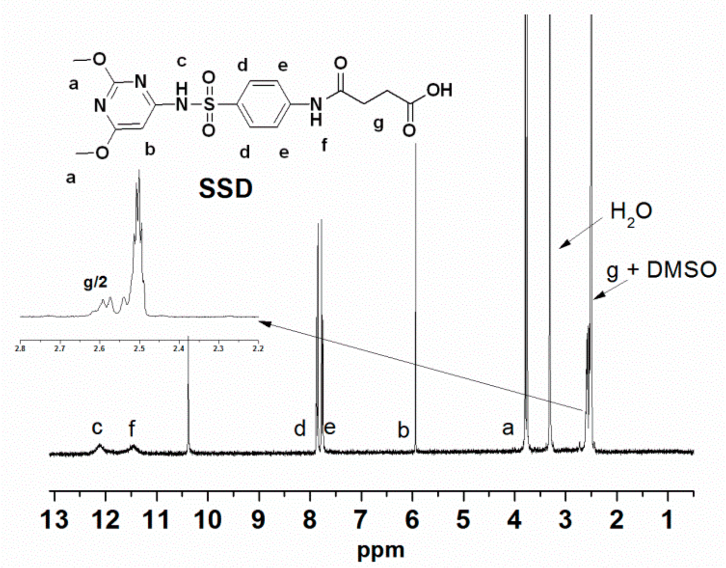

Figure S1. ^{1}H NMR spectra of SSD in DMSO-*d6*.

N-epsilon-carbobenzyloxy-L-lysine-*N*-carboxyanhydride (Lys(z)–NCA) was synthesized via triphosgene and *N*-epsilon-carbobenzyloxy-L-lysine (Lys(z)) in anhydrous THF. Briefly, Lys(z) and triphosgene at the molar ratio of 3:1.1 were added to THF, and then stirred till transparent at 60 °C. The white solid obtained by precipitating to anhydrous light petroleum and further purified by recrystallization in light petroleum and ethyl acetate.

PEG–PLL diblock copolymer was synthesized by ring opening polymerization of Lys(z)–NCA initiated by PEG-NH_2 and then de-protection of z-groups. PEG-NH_2 (0.4 mmol) and Lys(z)–NCA (8 mmol) were dissolved in 100 mL of anhydrous $CHCl_3$ at 30 °C and stirred for 72 h. The polymer was precipitated by pouring the cooled solution in an excess amount of diethyl ether with vigorous stirring and filtrated. The white solid product PEG–PLys(z) was dried in vacuo at room temperature for several days. The z-groups of PEG–PLys(z) were eliminated by HBr in trifluoroacetic acid at room temperature, and produced PEG–PLL.

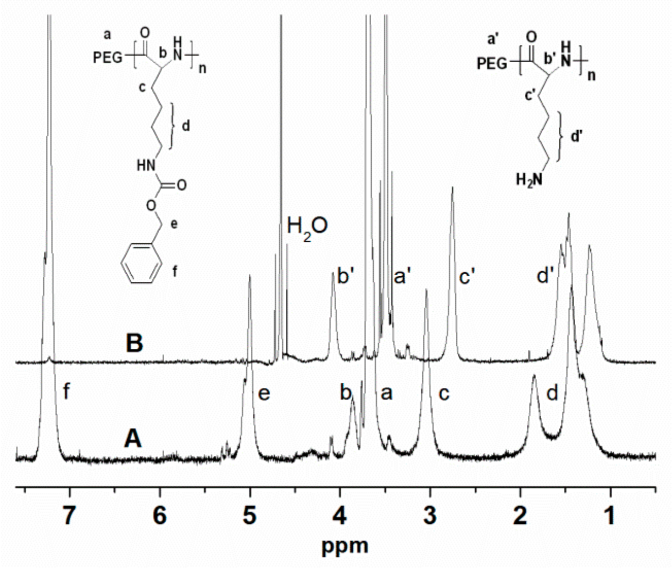

Figure S2. ^{1}H NMR spectra of PEG-PLys(z) in TFA-*d* and PEG-PLL in D_2O.

For the grafting reaction of PEG–PLL with SSD, the SSD acid group was activated in 50 mL of DMF by nhydroxysuccinicimide (HO-Su) and dicyclohexylcarbodiimide (DCC) with the molar ratio of SSD:HO-Su:DCC = 1:2:2. SSD (2 mmol) was dissolved in 30 mL of DMF and the mixture stirred at room temperature, and NHS (4 mmol) was added. After 1 h, DCC (4 mmol) was added to the reaction mixture and the mixture was stirred for 12 h. After removal of dicyclohexylurea by filtration, the reaction mixture was precipitated in diethyl ether twice and dried in vacuo overnight. Activated (1.2 mmol) was dissolved in 30 mL DMF at room temperature. PEG-PLL (0.04 mmol) was then added in the reaction mixture. The grafting reaction was carried out for 72 h at room temperature. The contents were poured into diethyl ether-ethanol component solvent to precipitate the product and dried *in vacuo*.

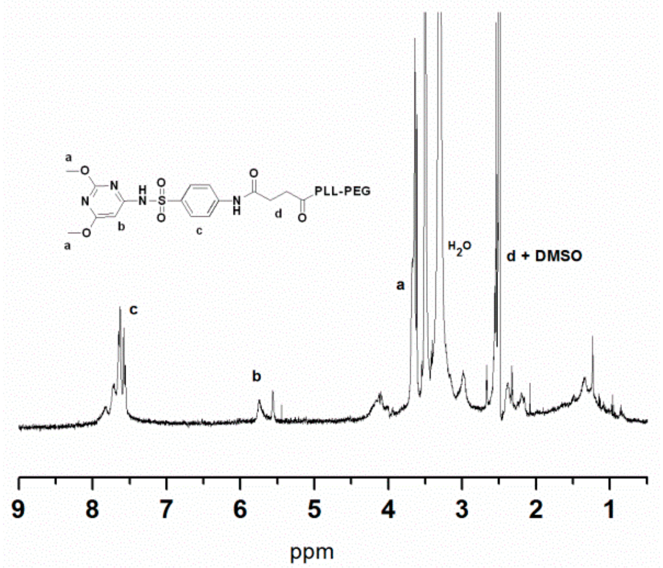

Figure S3. ^{1}H NMR spectra of PPSD in DMSO-*d6*.

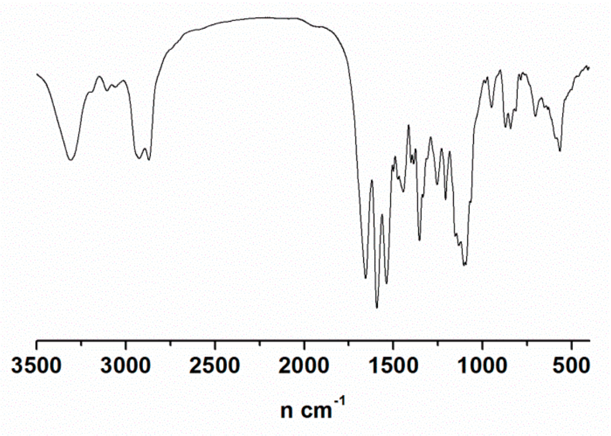

Figure S4. FT-IR spectra of PPSD in KBr.

The synthesized materials were characterized by FT-IR (Bruker Vertex 70, Karlsruhe, Germany) and NMR (Bruker AM-300, Karlsruhe, Germany).

© 2016 by the authors; licensee MDPI, Basel, Switzerland. This article is an open access article distributed under the terms and conditions of the Creative Commons by Attribution (CC-BY) license (http://creativecommons.org/licenses/by/4.0/).
